# Detection and correction of regional shape bias arising from imaging protocol: differences between GRE and SSFP

**DOI:** 10.1186/1532-429X-14-S1-P270

**Published:** 2012-02-01

**Authors:** Pau Medrano-Gracia, David A Bluemke, Brett R Cowan, J Paul Finn, Daniel C Lee, Joao A Lima, Avan Suinesiaputra, Alistair A Young

**Affiliations:** 1 Auckland Bioengineering Institute, The University of Auckland, Auckland, New Zealand; 2 National Institute of Biomedical Imaging and Bioengineering, NIH Clinical Center, Bethesda, MD, USA; 3 Diagnostic CardioVascular Imaging, UCLA, Los Angeles, CA, USA; 4 D. W. R. Cardiovascular Clinical Research Center, Johns Hopkins University, Baltimore, MD, USA; 5 Division of Cardiology, Northwestern University, Chicago, IL, USA

## Summary

We present a methodology to detect and correct shape bias arising from imaging protocol in an atlas of the left ventricle. We show how it can be used to correct the differences between GRE and SSFP shapes and volumes.

## Background

It is well established that different imaging protocols lead to different clinical parameters, for example gradient recalled echo (GRE) and steady state free precession (SSFP) protocols lead to different cardiac mass and volumes [1] . However, it is not known whether these differences are local or global in nature. Correction of local biases in heart shape is required for patient follow-up and meta-analyses of clinical trials which acquire data using different protocols.

For example, the Cardiac Atlas Project (CAP) [2] includes GRE images of asymptomatic volunteers from the MESA study [3] and patients with myocardial infarction imaged by SSFP from the DETERMINE study [4]. By computing a shape mapping from one protocol to another, we can establish a bias-free comparison between these two populations.

## Methods

Finite element shape models of the left ventricle (LV) were customized to 46 normal volunteers imaged by both GRE and SSFP and a multi-dimensional statistical transform was developed to correct the systematic bias between the protocols. Given that in our method there is no *a priori* guarantee that the mapping of local parameters will also obey the previously reported differences in terms of mass and volumes, errors were examined using both local shape and global volumetric measures.

## Results

**Figure 1:**
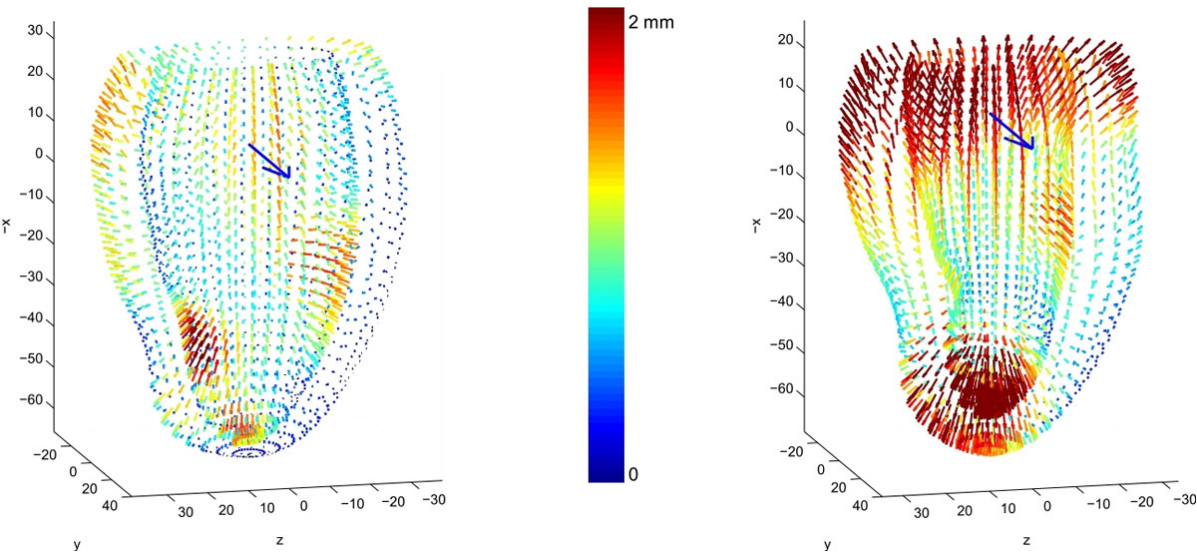
Shape bias between GRE and SSFP at ED (left) and ES (right); the arrow points from the centre toward the septum.

There was a systematic local difference between GRE and SSFP around the apex and papillary muscles at ED, and also around the base at ES (Fig. 1) which is probably due to the flow induced variation in blood-myocardial contrast in GRE imaging. The bias was corrected locally and the mapping also provided a global correction for volume (Table 1). Further, cross-validation experiments showed that this methodology only required ~25 cases with both modalities to determine the transformation to robustly convert between scanning techniques.

**Table 1:** Volume comparison of the original volumes between GRE and SSFP and the estimated ones from the leave-one-out experiments (L1). The error in volume is computed using the SSFP volume as reference.

Cavity vol. ED (ml)	GRE	SSFP	Estimated SSFP (L1)
Mean (μ)	126.2	134.8	135.6
Std. dev. (σ)	27.0	28.4	29.0
Error mean	-8.6	--	0.9
Error std. dev.	12.4	--	12.9
Cavity vol. ES (ml)			
Mean (μ)	52.8	53.5	54.3
Std. dev. (σ)	12.6	13.8	13.4
Error mean	-0.6	--	0.9
Error std. dev.	8.2	--	8.8
LV mass ED (g)			
Mean (μ)	145.3	132.3	130.9
Std. dev. (σ)	33.1	32.1	30.7
Error mean	12.34	--	-1.2
Error std. dev.	10.16	--	11.1

This methodology can also be successfully applied to other types of bias, for instance, that of different analysts or, between data processed by different laboratories.

## Conclusions

GRE and SSFP cardiac imaging protocols give rise to regional differences in LV geometry. We have established a generalisable framework which removes these biases and also corrects for mass and volumes.

## Funding

NIH - National Heart, Lung and Blood Institute (Award Number R01HL087773).
